# Transcriptome profilings of two tall fescue (*Festuca arundinacea*) cultivars in response to lead (Pb) stress

**DOI:** 10.1186/s12864-016-3479-3

**Published:** 2017-02-10

**Authors:** Huiying Li, Tao Hu, Erick Amombo, Jinmin Fu

**Affiliations:** Key Laboratory of Plant Germplasm Enhancement and Specialty Agriculture, Wuhan Botanical Garden, The Chinese Academy of Sciences, Lumo street, Wuhan City, Hubei 430074 People’s Republic of China

**Keywords:** Tall fescue, Pb stress, RNA sequencing, Terpenoid and polyketide metabolism, Transport and catabolism

## Abstract

**Background:**

Lead (Pb) is one of the most toxic heavy metal environmental pollutants. Tall fescue is an important cold season turf grass which can tolerate and accumulate substantial amount of Pb. To estimate genes related to Pb response and the molecular mechanism associated with Pb tolerance and accumulation, we analyzed the transcriptome of tall fescue in response to Pb treatment.

**Results:**

RNA-sequencing was performed in two tall fescue cultivars, Pb tolerant Silverado and Pb sensitive AST7001. A total of 810,146 assembled unique transcripts representing 25,415 unigenes were obtained from the tall fescue leaves. Among the panel, 3,696 differentially expressed genes (DEGs) were detected between the Pb treated (1000 mg/L) and untreated samples. Gene ontology (GO) and pathway enrichment analysis demonstrated that the DEGs were mainly implicated in energy metabolism, metabolism of terpenoids and polyketides, and carbohydrate metabolism related pathways. The expression patterns of 16 randomly selected genes were in consistent with that from the Solexa analysis using quantitative reverse-transcription PCR. In addition, compared to the common transcriptional response to Pb stress in both cultivars, the regulation of numerous genes including those involved in zeatin biosynthesis, limonene and pinene degradation, phagosome was exclusive to one cultivar.

**Conclusions:**

The tall fescue assembled transcriptome provided substantial molecular resources for further genomics analysis of turfgrass in response to heavy metal stress. The significant expression difference of specific unigenes may account for Pb tolerance or accumulation in two different tall fescue cultivars. This study provided new insights for the investigation of the molecular basis of Pb tolerance and accumulation in tall fescue as well as other related turf grass species.

**Electronic supplementary material:**

The online version of this article (doi:10.1186/s12864-016-3479-3) contains supplementary material, which is available to authorized users.

## Background

Lead (Pb) pollution is a severe global challenge due to it harmful effects on the environment, plant, and even human beings. The Pb pollution sources can be derived from various anthropogenic activities, such as mining and smelting activities, exhaust fumes of automobiles, industrial emissions, and applications of Pb-containing chemical materials including paints, gasoline, explosives, agrochemical and fertilizers [1]. As one of the most toxic non-essential elements, Pb is readily absorbed by plant roots, then eventually transferred and accumulated in different tissues. Excess Pb in plants often results in unprecedented adverse effects, including seed germination inhibition, stunted growth of seedlings, chlorosis, and a remarkable decrease in crop productivity [2]. In addition, Pb can stimulate the generation of free radicals and reactive oxygen species, hence leading to oxidative stress and DNA damage in plant [3].

Recently, phytoremediation has been considered to be a promising technique to clean up the heavy metals and limit their bioactivities. This would be achieved by exploiting the super capacity of specialized plants to tolerate, translocate, and eventually accumulate inordinate amount of heavy metal elements in harvestable parts [4]. Currently, more than 450 plant species have been identified as heavy metal accumulators. However, Pb hyper-accumulators or accumulators species are remarkably rare [5]. In addition, Pb is mainly fixed in the root with only a little translocated to the shoots. Thus, the lack of Pb hyper-accumulators, their relatively low biomass [6], as well as the poor adaptation ability to diverse environmental conditions [7] severely hamper the phytoremediation of Pb contaminated soils.

Tall fescue (*Festuca arundinacea* Schreb), belonging to the family poaceae, is an economically important cool season turfgrass and forage that is widely planted in temperate zones. Tall fescue is also a hexaploid outcrossing species with a large genome size [8], and up to now its genome has not been sequenced. As a long-lived perennial bunchgrass species, tall fescue is tolerant to various abiotic stresses, and can grow vigorously in a wide range of soil and climatic conditions. More interestingly, tall fescue can tolerate and accumulate substantial amount of heavy metals, especially Pb. It was earlier reported that tall fescue could grow in heavy metal contaminated sewage sludge [9]. Later, it was confirmed that tall fescue can absorb large amount of Pb when cultivated in Pb polluted solutions or soils without displaying any Pb intoxication symptoms [10, 11]. Some chelates including EDTA and acetic acid could significantly increase the accumulation of Pb in shoot, hence resulted in a higher Pb translocation index [12]. Emphatically, the biomass was not statistically affected by moderate Pb [13]. Our recent study indicated that there were significant differences in Pb tolerance and accumulating capacity among different tall fescue genotypes [14]. We noted that AST7001 could absorb a large amount of Pb, but was relatively sensitive to high concentration of Pb. Whereas, Silverado was tolerant to Pb, but could accumulate a little of Pb [14]. Variable physiological and gene expressional responses to Pb treatment were observed in Pb tolerant Silverado and Pb sensitive AST7001, respectively [15]. Consequently, due to the large biomass and the superior ability to accumulate large amounts of Pb, specific tall fescue genotypes can be screened and used as potential accumulator species for remediation of Pb contaminated environment. However, up to date, molecular mechanism of tall fescue responses to Pb stress is still unknown.

Studies have suggested that stress responses were controlled by a range of gene regulatory mechanisms that could act collectively under a specific stress. Plants could sense metal ions signals, activate copious metal transporters, and regulate particular transcription factors, and eventually counteracting and detoxifying the excessive heavy metals in their surroundings [16]. Previous studies in Arabidopsis identified many transporters which function in Pb detoxification [17]. In addition, HMA3, a P-type ATPase, was able to improve Pb tolerance by sequestrating Pb in the vacuoles of Arabidopsis [18, 19]. Besides, in Arabidopsis, *AtMRP3* and *Ethylene-Insensitive 2* (*EIN2*) genes were equally necessary for Pb resistance [20]. Chelation is considered to be another crucial mechanism that modulates Pb tolerance in Arabidopsis. Numerous micro molecules involved in metallothioneins, phytochelatins and glutathione were activated under Pb treatments [21, 22]. Moreover, genes related to some specific pathways, such as sulfur assimilation, indoleacetic acid and jasmonic acid biosynthesis were also induced by Pb stress [23]. Although these studies characterized some genes involved in Pb detoxification, many questions concerning their regulation mode and signaling networks are yet to be answered.

Through the past decade, next-generation sequencing based on RNA-seq has been employed as a powerful high throughout method for exploring the gene expression variation and regulation networks in various species responding to Pb stresses. Shen et al., [24] employed RNA-seq to analyze transcriptomic changes in maize (*Zea mays* L.) under Pb treatment or normal conditions. The genomic expression profile analysis revealed important transcripts in maize roots responding to Pb stress. More recently, Gao et al. [25] analyzed the maize root transcriptome of a non-hyperaccumulator inbred line 9782 under Pb pollution by RNA-Seq method. As a result, many transcription factor families, including bZIP, ERF and GARP, were found to act as the important regulators at different developmental stages when exposed to Pb stress. In addition, using RNA-seq technology, Wang et al. [26] analyzed the transcriptome profiling of radish (*Raphanus sativus* L.) root, and identified many genes involved in Pb Stress. However, the transcriptional alteration in tall fescue under Pb stress has not been reported.

In the present study, two tall fescue genotypes, Pb tolerant Silverado and Pb sensitive AST7001 identified in our previous study [15], were selected to explore the Pb stress responses at transcriptional level using RNA-Seq. Thus, the objectives of this study were to document the initial alternation profile of transcriptome at genome-wide level; to explore the critical genes or pathways involved in regulating tall fescue tolerance to Pb; and finally to facilitate further investigation of the underlying mechanisms of Pb accumulation or tolerance in plants.

## Results and discussion

### Analyses of RNA-Seq data

From our previous studies, we observed that AST7001 shoots could accumulate remarkably more Pb (19.92 mg g^−1^ dry wt) than Silverado (1.43 mg g^−1^ dry wt) after 48 h of Pb treatment (1000 mg L^−1^) [14, 15]. To investigate the genes involved in Pb stress response or Pb accumulation in different tall fescue cultivars, a set of cDNA libraries were constructed from total RNA isolated from leaves with or without Pb treatments. The libraries were then sequenced using the Illumina HiSeq 2000 platform. An overview of the RNA-Seq reads derived from the sequencing was presented in Table 1. Totally, approximately 141 million and 137 million raw reads as well as 131 million and 128 million clean reads were obtained from Silverado and AST7001, with an average GC content of 47.29% and 47.08%, respectively (Table 1). In addition, the higher Q20 (97.74% and 97.87 in Silverado and AST7001, respectively), and Q30 value (93.69% for both cultivars) indicated the high quality of the transcriptome sequencing (Table 1).

**Table 1:** Overview of the sequencing results

Sample name	Raw reads	Clean reads	Q20(%)	Q30(%)	GC(%)
Silverado 0 h	39,316,448	36,688,349	97.74	93.79	47.73
Silverado 4 h	49,698,475	46,353,176	97.74	93.78	47.57
Silverado 48 h	51,518,056	48,012,261	97.75	93.5	46.57
Subtotal/average	140,532,979	131,053,786	97.74	93.69	47.29
AST7001 0 h	47,372,198	44,083,453	97.64	93.18	47.79
AST7001 4 h	43,276,947	40,527,394	97.91	93.79	46.79
AST7001 48 h	46,050,140	43,344,206	98.07	94.1	46.67
Subtotal/average	136,699,285	127,955,053	97.87	93.69	47.08

Leaves of two tall fescue cultivars with different Pb tolerance, Silverado (tolerant) and AST7001 (sensitive) were sampled for RNA sequencing after exposed to 0 (control) and 1000 mg/L Pb(NO)3 (Pb) for 4 and 48 h

Due to the absence of tall fescue genomic sequences, *de novo* assembly was carried out to construct transcripts from clean reads using Trinity software (http://trinityrnaseq.github.io), which has been considered to be an efficient method for de novo reconstruction of transcriptome from RNA-Seq data. As shown in Table 2, a total of 736,651 contigs were generated for Silverado, with the maximum length of 13,550 bp, the mean length of 243.39 bp and N50 of 270 bp. A total of 776,603 contigs were produced for AST7001, with the maximum length of 14,863 bp, the average length of 246.16 bp and N50 of 278 bp. By using the Trinity assembler, 397,445 transcripts were further obtained for Silverado, a maximum length of 13,607 bp, the mean length of 774 bp and an N50 of 1261 bp. Conversely, 412,701 transcripts were generated for AST7001, with a maximum length of 14,472 bp, average length of 770 bp and N50 of 1,254 bp. Finally, from these transcripts, 25,415 unigenes were assembled with an average length of 1450 bp, N50 of 2048 bp, and the maximum length of 14,472 bp. All the unigenes were listed in Additional file 1. The mean length of the ungenes in present study (1450 bp) was slightly shorter than the previously reported in tall fescue (1840 bp) [27], which might be attributed to the genotypic differences. However, it was profoundly longer than those in other plants, including bermudagrass (*Cynodon dactylon*; about 700 bp) [28, 29], tea (*Camellia sinensis*; 355 bp) [30], and litchi (*Litchi chinensis* Sonn; 601 bp) [31].

**Table 2:** Summary of the RNA-Seq data from the Trinity de novo assembly program in tall fescue leaves

Assembly statistic (Contig)	Accession name	Total Length (bp)	Sequence No.	Max Length (bp)	Ave Length (bp)	N50	>N50 Reads	GC%
	Silverado	179,294,448	736,651	13,550	243.39	270	140,457	45.36
	AST7001	191,169,458	776,603	14,863	246.16	278	145,695	45.33
Assembly statistic (Transcript)		Total Length (bp)	Transcript No.	Max Length (bp)	Ave Length (bp)	N50	>N50 Reads	GC%
	Silverado	307,585,704	397,445	13,607	774	1,261	72,603	47.47
	AST7001	317,664,615	412,701	14,472	770	1,254	75,095	47.46
Unigene		Total Length (bp)	Unigene No.	Max Length (bp)	Ave Length (bp)	N50	>N50 Reads	GC%
		36,859,338	25,415	14,472	1,450	2,048	6,050	48.29

The transcriptome profiling of radish (*Raphanus sativus* L.) and maize root response to Pb treatments using RNA-seq technology had been performed previously [24, 26], and many unigenes involved in response to Pb stress had been identified. In tall fescue, Hu et al. [27] investigated the unigenes associated with high temperature response and detected a many differentially expressed genes through next generation sequencing. Despite the prominent Pb tolerance and Pb accumulation in tall fescue, the molecular mechanisms involved in Pb response were markedly unkown. Therefore, the unigenes obtained in this study could greatly enrich the data of trancriptomes responsive to heavy metal stress in tall fescue, and provide useful information for other turfgrass and forage grass.

### GO annotation of the tall fescue transcriptomes

**Fig. 1:**
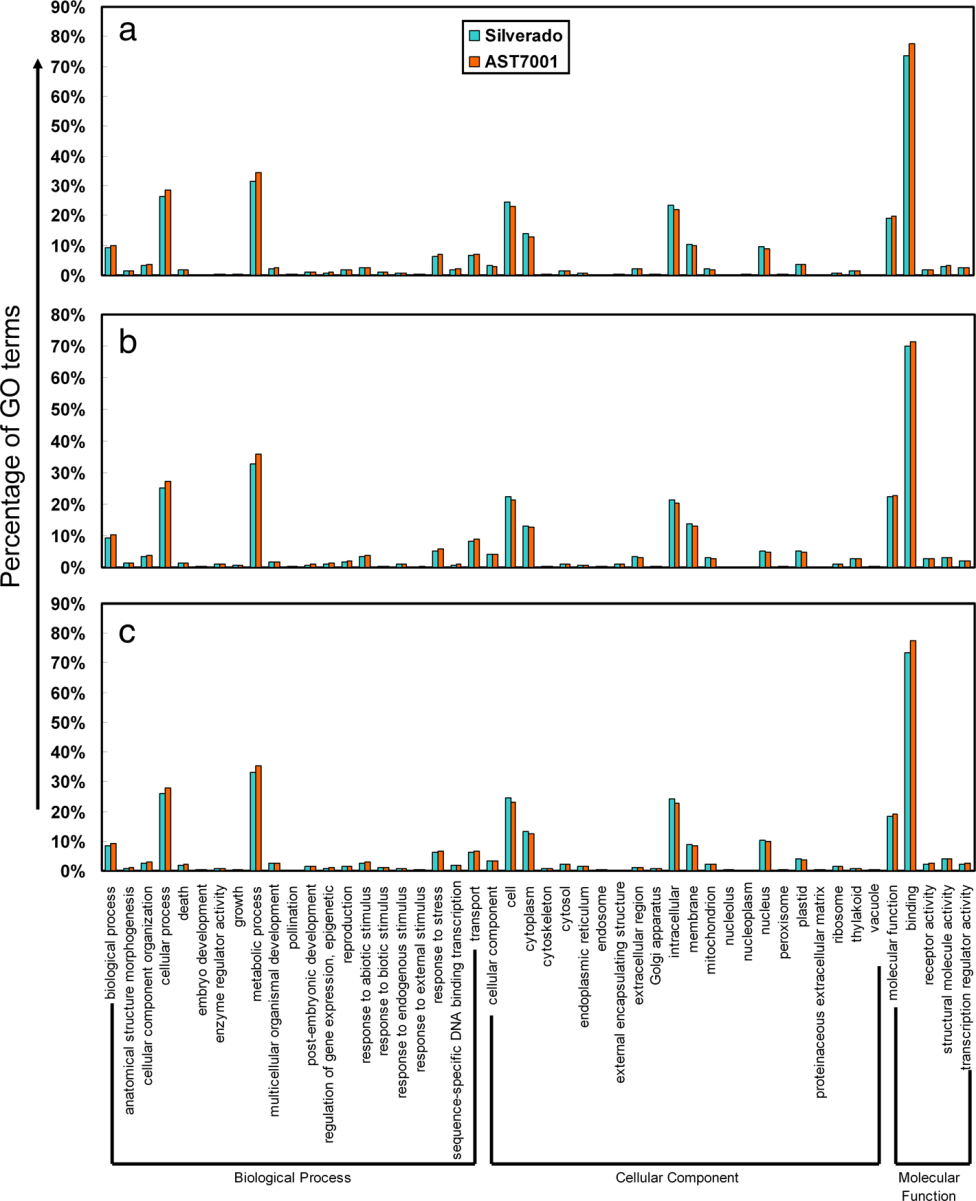
Gene Ontology (GO) classification of assembled unigenes in two tall fescue cultivars, Pb-tolerant Silverado and Pb-sensitive AST7001. **a** Functional categorization of two cultivars under control condition; (**b**) Functional categorization after 4 h of Pb treatment; (**c**) Functional categorization after 48 h of Pb treatment. The unigenes were summarized in three main GO categories (biological process, cellular component and molecular function) and 48 subcategories. The x-axis indicated the subcategories, while the y-axis indicated the percentage of each subcategory in their category

According to the GO analysis (http://www.geneontology.org), among 25,416 unigenes, 18,326 (72.1%) were classified into at least one GO term under three ontologies: biological process, cellular components and molecular function (Fig. 1). Totally, the GO classification patterns in the two tall fescue cultivars were similar under normal condition or Pb treatments, despite little differences among specific categories. Within biological process ontology, unigenes involved in ‘metabolic process’ (above 31%), ‘cellular process’ (above 26%), and ‘biological process’ (above 9%) were highly represented. In addition, the unigenes related to ‘response to stress’ was also relatively abundant. Notably, the proportion of ‘transport’ category was increased from 6.51% to 8.16%, as well as 7.11% to 8.89% in Silverado and AST7001, respectively, after 4 h of treatment by Pb when compared to the normal condition. With the extension of Pb treatment, the proportion of ‘transport’ category declined to the initial level. The cellular component ontology mainly comprised genes associated with ‘cell’ (above 20%), ‘intracellular’ (above 20%), and ‘cytoplasm’ (above 10%). While in terms of molecular function ontology, ‘binding’ (above 70%) and ‘molecular function’ (above 18%) were the highly represented.

The GO annotation result in this study was remarkably different from that of radish, particularly in terms of the molecular function ontology [26], which might be attributed to the species difference. However, our result was consistent with that from tall fescue in response to heat stress [27], suggesting that there might be some common responsive mechanisms in tall fescue under different abiotic stress. In addition, the variance of different categories under three ontologies was similar along with the extension of Pb treatment. For example, at 4 h of Pb treatment, genes involved in ‘vacuole’ category under cellular component GO ontology was more than twice abundant than in normal condition for both cultivars. When the treatment time extended to 48 h, the proportion of ‘vacuole’ returned to the normal level. The category of ‘response to abiotic stimulus’ exhibited the same variation tendency among the two tall fescue cultivars. However, despite the similarity, there was obvious variation of GO annotation between two cultivars. Genes encoding for ‘metabolic process’, ‘cellular process’ and ‘binding’ related proteins were more dominant in Pb sensitive AST7001 than in Pb tolerant Silverado, under each condition. On the contrary, the categories of ‘cellular’ and ‘intracellular’ ‘binding’ showed a reverse trend (Fig. 1). These differences might be the molecular foundation for the variance of Pb stress tolerance or Pb accumulation.

### COG functional annotation and classification

**Fig. 2:**
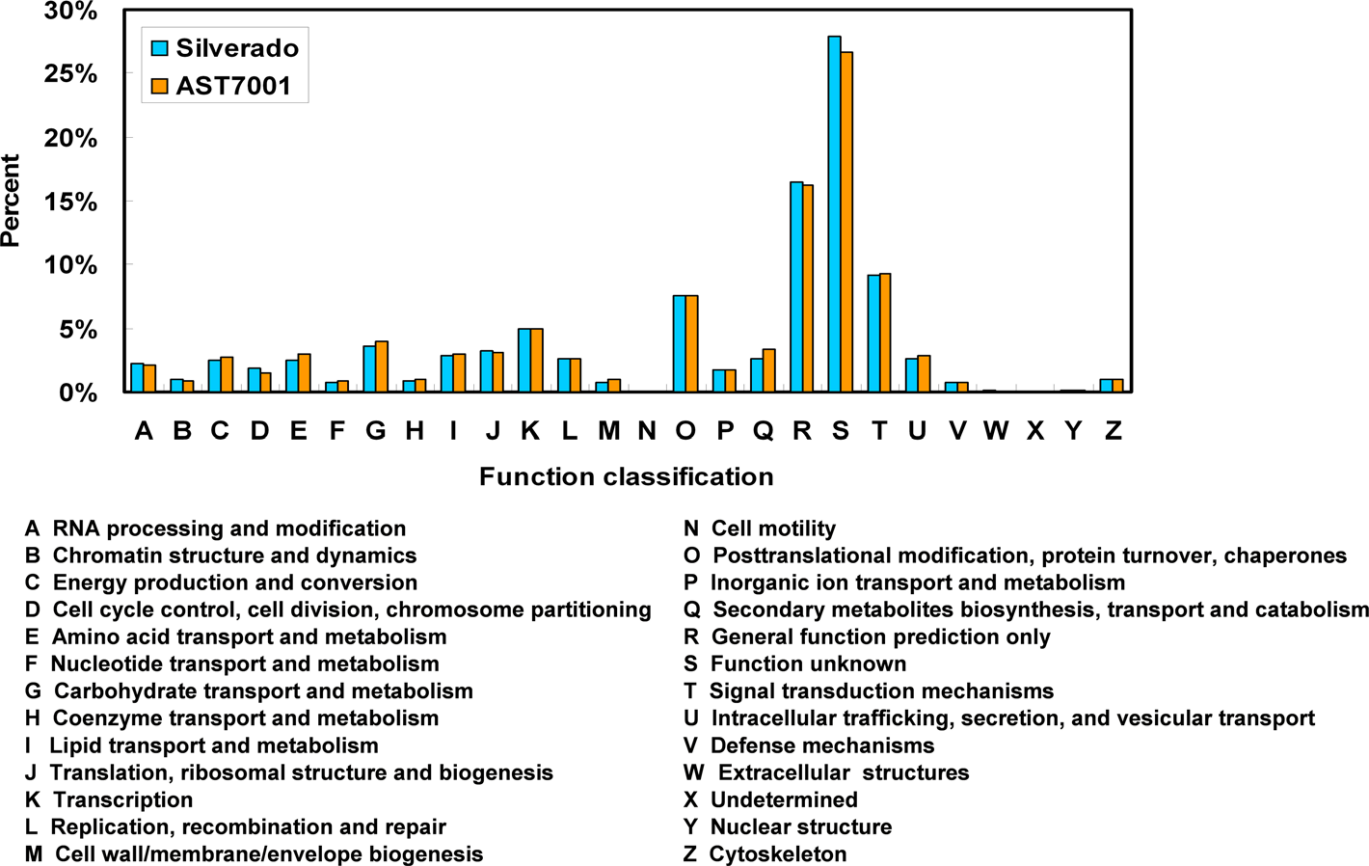
Non-supervised Orthologous Groups (eggNOG) classification. For each cultivar, all assembled unigenes from the two tall fescue cultivars were aligned to the Clusters of Orthologous Groups (COG) and Eukaryotic Orthologous Group (KOG) database to predict and classify possible functions

To evaluate the distribution characteristics in gene function of the *de novo* assembled transcriptome, all unigenes of the two tall fescue cultivars were subjected to Clusters of Orthologous Groups (COG) classification for functional prediction. As a result, a total of 18,570 (8716 for Silverado, 9854 for AST7001, respectively) unigenes were clustered into 26 COG classifications (Fig. 2), accounting for 73.1% of the total unigenes (25, 416). Although there were more unigenes assigned to COG classification in AST7001 than in Silverado, the distribution pattern of these unigenes in the COG categories was very similar between two cultivars. Among all COG categories, the ‘function unknown’ (27.91%; 26.63%) and ‘general function prediction only’ (16.46%; 16.24%) were the biggest two clusters for Silverado and AST7001. The next five main categories were ‘signal transduction mechanisms’ (9.22%; 9.30%), ‘posttranslational modification, protein turnover, chaperones’ (7.56%; 7.51%), ‘transcription’ (5.01%; 4.97%), ‘carbohydrate transport and metabolism’ (3.64%; 3.96%), ‘translation, ribosomal structure and biogenesis’ (3.26%; 3.12%) for both cultivars. On the contrary, only a few unigenes were assigned to ‘Extracellular structures’ (totally 12 unigenes), ‘cell motility’ (totally 8 unigenes) or ‘undetermined’ (just 1 unigene for Silverado). This result was consistent with that of Hu et al. [27] findings on tall fescue exposed to high temperature, who also observed that the cluster of ‘function unknown’ (27.55%) and ‘general function prediction only’ represented the biggest two groups, while ‘cell motility’ and ‘undetermined’ comprised very few unigenes, indicating the common characteristics of tall fescue species.

In radish response to Pb [26], unigenes were grouped only into 23 COG functional categories, with the five largest categories of ‘general function prediction only’ (30.05%), ‘signal transduction mechanisms’ (15.89%), ‘transcription’ (14.70%), ‘post translational modification, protein turnover, chaperones’ (13.59%), and ‘function unknown (12.69%). This result was consistent with ours. However, there were more unigenes clustered in ‘function unknown’ in tall fescue compared with radish. This could be attributed to the lack of genomic information of tall fescue, and the COG analysis was performed against databases from five gramineae plants, including Brachypodium hereafter (*Brachypodium distachyon*), rice (*Oryza sativa* Linn.), maize, wheat (*Triticum* spp.), and sorghum [*Sorghum bicolor* (L.) Moench].

### Differential expression of assembled transcripts

**Fig. 3:**
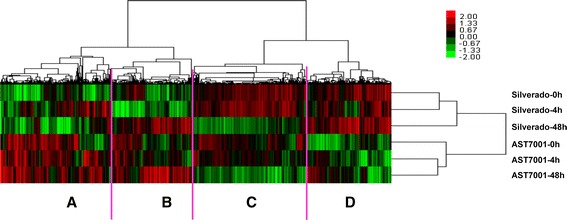
Hierarchical clustering analysis of Pb-induced changes in gene expression in two tall fescue cultivars under different conditions. *Red* and *green* colors in the heat-maps represented up-regulated and down-regulated genes, respectively; while *black* color represented those genes showing no changes. Scale bar denoted the value of fold change

Normalized RPKM (reads per kilobase per million) was employed to quantify the abundance of each assembled unigene, and to compare the mRNA levels among different cultivars or time points after Pb treatments. Those unigenes that were significantly abundant or rare in one cultivar and/or time point relative to the other cultivar and/or time point (q-value < 0.05 and | fold change| >2) were defined as differentially expressed genes (DEGs). By using Cluster 3.0 software, the resulting DEGs from the two cultivars under different treatment time were clustered and were clearly separated according to the hierarchical clustering (Fig. 3). From Fig. 3, all the DEGs were clustered into four groups with distinct expression profiles. Most of the DEGs showed up-regulation in group B, while down-regulation in a large group C for both cultivars under 48 h of Pb treatment, when compared to the control and 4 h of Pb treatment. Basically, in AST7001 most DEGs in Group A showed no remarkable differences in expression profiles between control and Pb treatments. Comparatively, after 4 h of Pb treatment, many DEGs in Group A exhibited up-regulation in Silverado. In group D, most DEGs showed no significant differences for each cultivar between control and Pb treatments. However, it was noticeable that group A and D displayed contrasting expression pattern between the two cultivars. For AST7001, most DEGs in group A were in high abundance, while for Silverado almost all of the DEGs in group D were in high abundance. This result suggested that the Pb tolerance variation between Silerado and AST7001 might be modulated by multiple genes, and the sampling time was suitable to acquire the target DEGs.

**Fig. 4:**
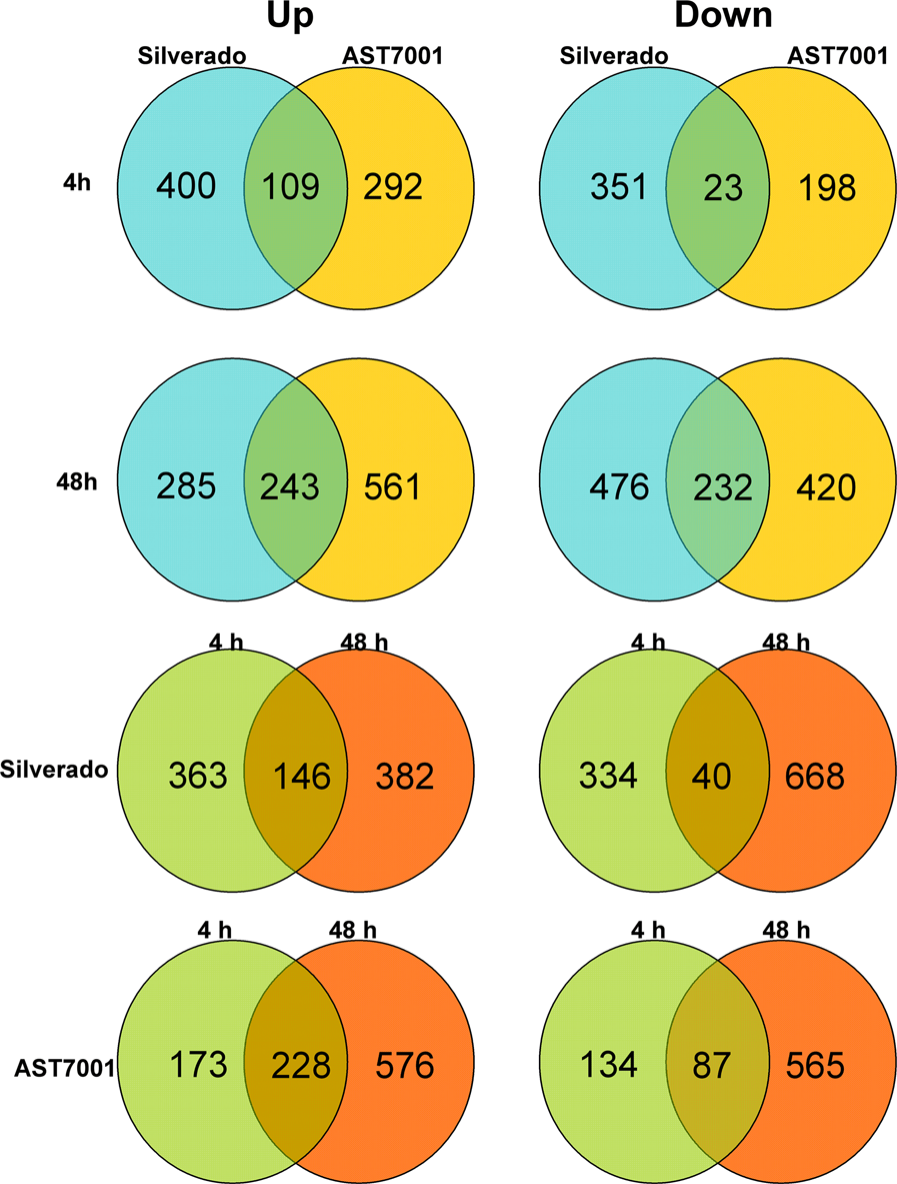
Venn diagram of differentially expressed genes (DEGs) (statistically significant >2-fold, *P <* 0.05, RPKM > 3). The sum of the numbers in each circle represented total number of DEGs; while the overlap part of the circles represents common DEGs between comparisons

Venn diagram analysis was performed to demonstrate both the common and specific DEGs between control and Pb-stressed plants in two cultivars. In total, more DEGs were found in Silverado (1933) than in AST7001 (1763), although the former displayed no visible phenotypic changes, while the latter exhibited slight dehydration. The result indicated that there were more common DEGs detected in both cultivars at 48 h than at 4 h of Pb treatment (243 versus 109 for up-regulated; 232 versus 23 for down-regulated; Fig. 4). For Silverado, the numbers of up-regulated genes were nearly the same after 4 h or 48 h of Pb treatment, whereas, the quantity of down-regulated genes was increased significantly from 4 h to 48 h of treatment (374 versus 708). For AST7001, the quantity of either activated or inhibited genes were both larger at 48 h than 12 h (804 versus 401 for up-regulated; 652 versus 221 for down-regulated). It was also observed that the number of common up-regulated DEGs was larger than that of common down-regulated DEGs, between the two Pb treatment groups for each cultivar. In addition, the number of DEGs increased linearly with the extension of Pb treatment time for both cultivars, indicating that Pb treatment on tall fescue could result in time course-dependent changes in gene expression. Totally, many DEGs were specific to a certain cultivar or one treatment group, and the common DEGs accounted for only a small portion.

The result suggested that the change of gene transcript level was an important response mechanism to heavy metal stress in tall fescue. Similarly, in previous study, Wang et al. [26] detected that 4,614 unigenes were differentially expressed between the two libraries of untreated (CK) and Pb-treated (Pb1000) roots in radish. Interestingly, more genes were modulated in Pb tolerant Silverado (883) than in sensitive AST7001 (622) at the early stage of Pb treatment, indicating that the tolerant cultivar activated gene expression more rapidly in response to Pb stress than the sensitive genotype. Chen et al. [29] reported that under cold stress, global gene expressions were initiated more rapidly in the cold-resistant bermudagrass genotype than those in the sensitive genotype, just consistent with this study. The rapid modulation of many genes may contribute to the complexity of abiotic tolerance in grass.

### Validation of the DEGs by qRT-PCR analysis

**Fig. 5:**
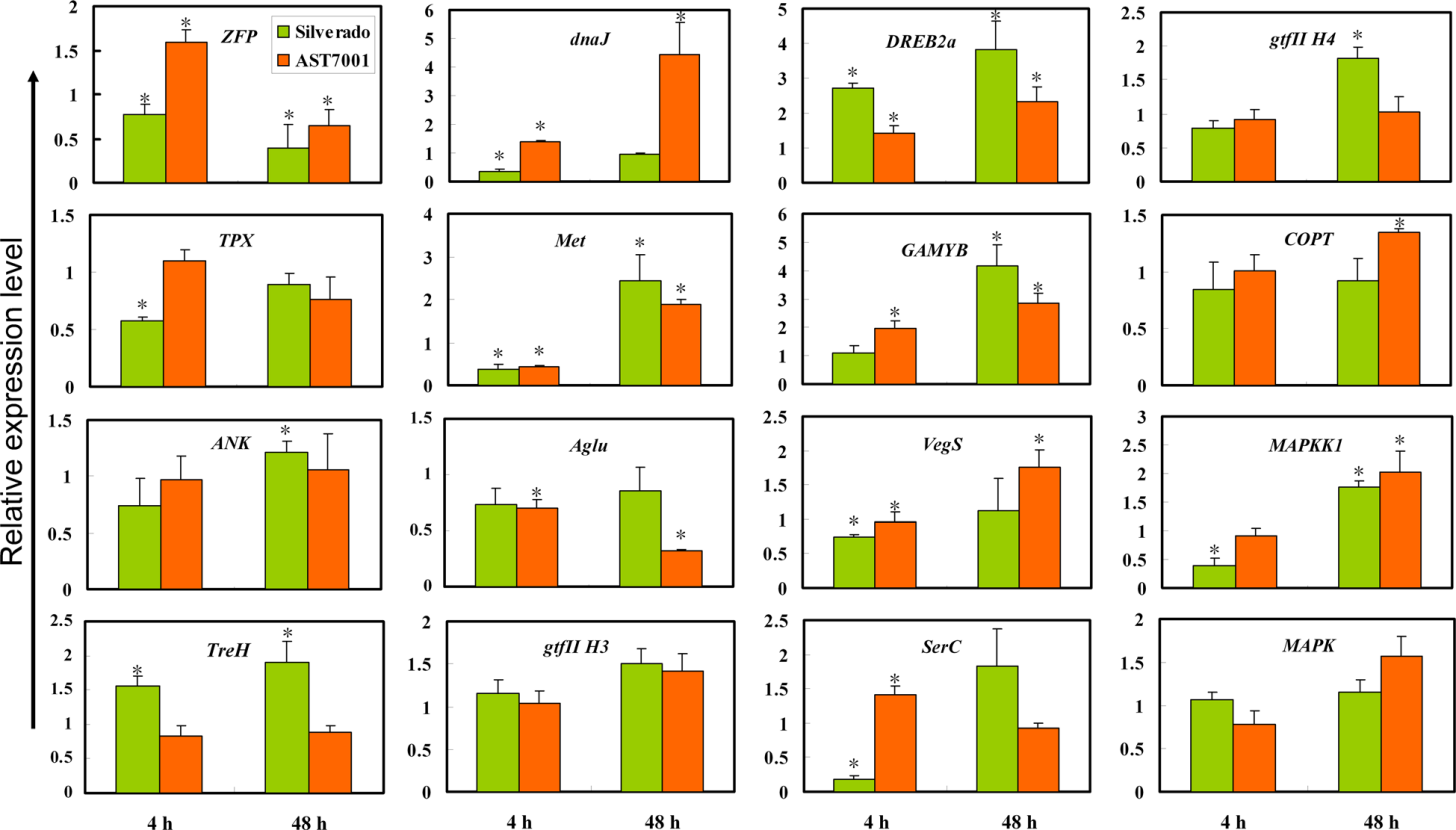
qRT-PCR analysis of sixteen selected DEGs in two tall fescue cultivars treated with Pb(NO_3_)_2_. *YT521-B* gene was used as the reference gene for normalization of gene-expression data. Three independent experiments and three technical replicates were performed. Asterisk indicated significant differences when compared to the control groups of each cultivar, respectively

To confirm the reliability of the Illumina RNA-Seq, 16 candidate unigenes involved in different biological process were selected and their expression in response to Pb stress was detected by quantitative reverse transcription-PCR (qRT-PCR) analysis. As result, three genes (*Met*, *DREB2a* and *ZFP*) were significantly regulated by Pb in both cultivars (Fig. 5). We observed that *DREB2a* maintained higher expression in Pb stressed plant, while *Met* was inhibited at 4 h, but up-regulated at 48 h of Pb treatment, compared to their respective controls. On the other hand, *ZFP* was suppressed in Pb stressed Silverado, but induced in AST7001 at 4 h. Four unigenes (*TPX*, *ANK*, *TreH* and *gtf IIH4*) were significantly regulated in Silverado at 4 h or (and) 48 h of Pb treatment, but displayed no transcriptional change in AST7001. By contrast, *Aglu* and *COPT* were significantly regulated in AST7001 at 48 h of Pb treatmen, but not affected in Silverado. In Silverado, *Vegs*, *SerC* and *MAPKK1* showed significantly decreased expression at 4 h of Pb treatment, respectively. However, these three genes were all up-regulated after 4 or 48 h of Pb treatment in AST7001. *GAMYB* was induced in both Pb stressed cultivars. After Pb treatment, *DnaJ* was significantly up-regulated in AST7001, but remarkably down-regulated in Silverado. The other genes showed no significant difference in both Pb stressed cultivars. All the primers used in this study were listed in Additional file 2.

**Fig. 6:**
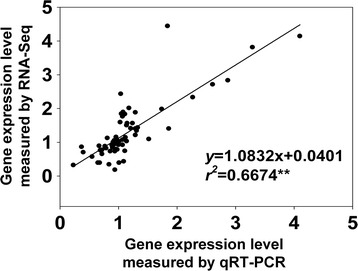
Correlations of expression level analyzed by RNA-Seq platform (y axis) with data resulted from qRT-PCR (x axis)

The correlation between the result form qRT-PCR and RNA-Seq was evaluated. As shown in Fig. 6, the results of qRT-PCR displayed strong correlation with that of RNA-sequencing (y = 1.0832× + 0.0401; *r* ^2^ = 0.6674; *P <* 0.0001), indicating that the RNA-Seq was accurate and effective.

### Gene Ontology (GO) enrichment and KEGG pathway enrichment analysis of DEGs

To understand the complex response to Pb stress in different tall fescue cultivars, it is very important to analyze which GO term is significantly overrepresented after stress. In present study, the hypergeometric distribution was used to determine whether a GO term was overrepresented on the background of the whole genome.

From Additional file 3, after 4 h of Pb treatment in Pb sensitive AST 7001, only two GO terms were significantly overrepresented (*P*-value < 0.05), i.e. ‘proteinaceous extracellular matrix’ of the ‘cellular component’ sub-ontology and ‘binding’ of ‘molecular function’ sub-ontology. When the treatment time extended to 48 h, three GO terms were significantly enriched, including ‘metabolic process’, ‘plastid’ and ‘thylakoid’. Conversely, in Pb tolerant Silverado, the GO enrichment patterns were different from that of AST7001. At 4 h of Pb treatment, there were only two GO terms that were significantly enriched, i.e. ‘death’ and ‘proteinaceous extracellular matrix’ (Additional file 3). However, when treated with Pb for 48 h, many GO terms were greatly overrepresented, including ‘thylakoid’, ‘plastid’, ‘extracellular region’, ‘membrane’, ‘cytoplasm’, ‘proteinaceous extracellular matrix’ and ‘ribosome’ under sub-ontology of ‘cellular component’; ‘metabolic process’, ‘response to abiotic stimulus’, ‘response to biotic stimulus’, ‘response to stress’, and ‘cellular component organization’ under sub-ontology of ‘biological process’; as well as ‘structural molecule activity’ under sub-ontology of ‘molecular function’.

The number of DEGs in each KEGG pathway was also estimated. After then, the hypergeometric distribution was calculated to determine which pathway was significantly enriched, compared to the background of whole genome. It was found that two KEGG pathways related to ‘metabolism of terpenoids and polyketides’ and ‘sensory system’ were significantly enriched in both cultivars at 4 h of Pb treatment versus control (*P*-value < 0.05) (Additional file 4). When the treatment time extended to 48 h, more pathways were significantly enriched in both cultivars. In Pb tolerant Silverado, most of pathways involved in metabolism were highly overrepresented, including ‘Energy metabolism’ (*P*-value < 0.0001), ‘carbohydrate metabolism’, ‘metabolism of terpenoids and polyketides’, ‘metabolism of cofactors and vitamins’, ‘amino acid metabolism’, ‘metabolism of other amino acids’. In addition, KEGG pathway related to ‘signal transduction’ was also significantly enriched after 48 h of Pb treatment, when compared to the control (*P*-value < 0.01). Similarly, in Pb sensitive AST7001, several pathways related to metabolism were significantly enriched, such as ‘energy metabolism’, ‘metabolism of terpenoids and polyketides’, ‘carbohydrate metabolism’ and ‘amino acid metabolism’. Besides, ‘sensory system’ was moderately enriched after 48 h of Pb treatment (Additional file 4).

### Metabolism involved photosynthesis is affected by Pb stress

**Fig. 7:**
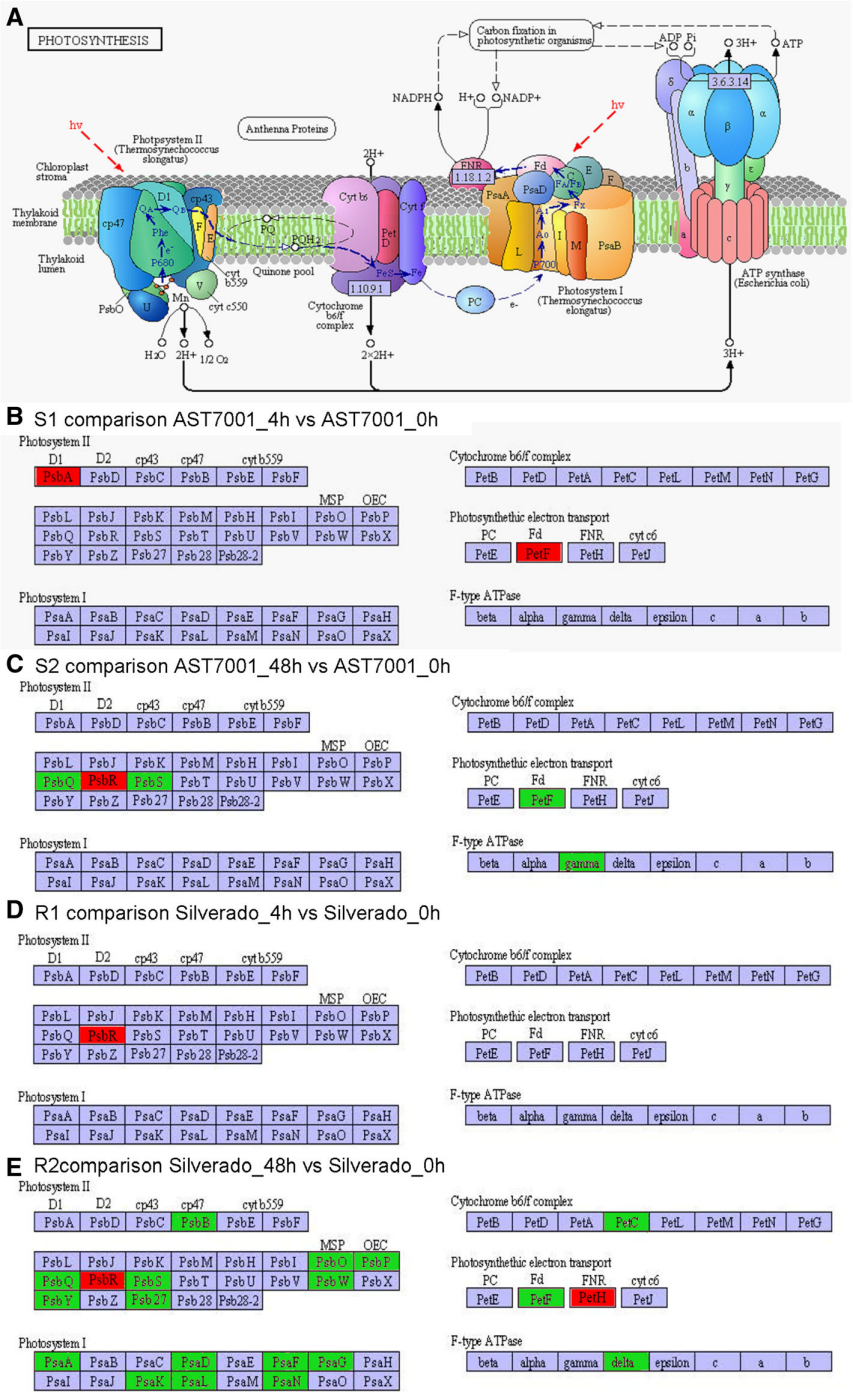
Differentially expressed genes (DEGs) in photosynthesis pathway in different combinations. Red denotes up-regulated genes and green denotes down-regulated genes. **a** showed the structure of thylakoid; **b**, **c**, **d** and **e** displayed DEGs in AST7001_4h vs AST7001_0h, AST7001_48h vs AST7001_0h, Silverado_4h vs Silverado_0h, and Silverado_48h vs Silverado_0h combinations, respectively

As a highly integrated and regulated process, and sensitive to environmental changes, photosythesis needs to balance light energy absorption by photosystems and the energy consumed by metabolic sinks [32]. However, abiotic stress may disrupt this balance, thereby necessitating photosynthetic adjustments to maintain the balance of energy flow [33]. In both tall fescue cultivars, the KEGG pathways related to energy metabolism was significantly enriched after Pb treatment for 48 h, especially those involved in photosynthesis assimilation pathway, such as ‘photosynthesis’, ‘photosynthesis-antenna proteins’, ‘carbon fixation in photosynthetic organisms’, ‘methane metabolism’ and ‘nitrogen metabolism’ (Additional file 5). The amount of DEGs involved in these pathways were 7, 5, 11, 4 and 5 transcripts for AST7001; and 22, 9, 17, 7 and 6 for Silverado, respectively. Almost all of the DEGs were down regulated in the two cultivars under Pb stress, except for 4 and 5 which were up-regulated in AST7001 and Silverado, respectively (Additional file 5). In addition 27 DEGs were annoted to the photosynthetic pathway. At 4 h of Pb treatment, only 2 and 1 DEGs were found to be up-regulated in AST7001 and Silverado, respectively, when compared to their controls. While at 48 h of Pb treatment, there were 1 up-regulated and 4 down-regulated DEGs in AST7001, as well as 2 up-regulated and 18 down-regulated DEGs in Silverado (Fig. 7, Additional file 5). We noted that more genes displayed down-regulation in Pb tolerant Silverado under stress. Consistent with our result, it was previously reported that under heat stress, there were more genes that were inhibited in heat tolerant tall fescue genotype than in the sensitive in the ‘photosynthesis-antenna proteins’ and ‘carbon fixation in photosynthetic organisms’ [27]. Similarly, in bermudagrass, it was also intriguingly observed that 23 photosynthesis related genes were all down-regulated by freezing temperature in cold resistant genotype without prior cold acclimation [29]. From these studies, despite the common phenomenon that photosynthesis was more severely affected in tolerant variety, the underlying mechanism for this phenomenon is still yet to be discovered.

In plant light-harvesting chlorophyll protein complexes (LHC), antenna proteins are very important for efficient absorption of light energy, serving as peripheral antenna systems [34]. In present study, all the DEGs involved in the photosynthesis-antenna proteins were down regulated in the two tall fescue cultivars after 48 h of Pb treatment (Additional file 5). Among which, 4 genes were related to LHCA, while 5 genes were involved in LHCB. The down regulation of these genes probably resulted in the decline of light energy absorption and subsequent photosynthesis. Consistent with this presumption, our previous study indicated that Pb treatment could decrease the value of Fv/Fm, a parameter generally used to indicate the maximum quantum efficiency of photosystem II, which is considered as a sensitive indication of plant photosynthetic performance, although the decrease degree was less in Silverado than in AST7001 [15]. Similarly, Pb in leaves could also lead to a decrease in Fv/Fm in other species, such as spinach [35], wheat [36] and *Ligustrum ovalifolium* [37]. In previous studies, the heavy metal stress was found to affect plant photosynthesis, as a result of the reduction of photosynthetic pigments in leaves [38]. More recently, Shen et al. [24] reported that KEGG pathways associated with photosynthesis, including photosynthesis-antenna proteins, carbon fixation in photosynthetic organisms, were also affected significantly in maize roots when exposed to Pb stress which is consistent with our result. These evidences suggested that the down regulation of photosynthesis pathway might be a common stress response to various Pb treatments among various plant species.

### Metabolism of terpenoids and polyketides were affected by Pb stress

Terpenoids represent a large group of secondary metabolites produced by plant with diverse structures. Their metabolites are involved in various basic functions in plant growth and development. Moreover, the majority of terpenoids, especially the volatile or semivolatile, including isoprene, monoterpenoids, sesquiterpenoids, and diterpenoids, are implicated in plant protection against abiotic stress and in various biotic interactions [39]. In addition, terpenoids also serve as antioxidants playing different roles in plants. Currently, three main ways of antioxidant action have been discovered for carotenoids. The antioxidant activity assay confirmed that gamma-terpinene was also a very effective antioxidant [40]. More recently, a study on transcriptome and metabolite profiling revealed that prolonged drought also modulated the terpenoid and phenylpropanoid pathway in white grapes (*Vitis vinifera* L.) [41].

In this study, the KEGG pathway related to the metabolism of terpenoids and polyketides in tall fescue was significantly enriched after Pb treatment in the two tall fescue cultivars (Additional file 5), and a total of 28 unigenes were differentially expressed. The common pathways responding to Pb included ‘terpenoid backbone biosynthesis’, ‘monoterpenoid biosynthesis’, ‘sesquiterpenoid and triterpenoid biosynthesis’, ‘diterpenoid biosynthesis’, ‘carotenoid biosynthesis’, ‘brassinosteroid biosynthesis’, and ‘zeatin biosynthesis’, involving 6, 2, 2, 3, 4, 1 and 2 differentially expressed transcripts for AST7001; and 5, 2, 1,3, 6, 1 and 2 for Silverado, respectively (Additional file 6). Both DEGs (TPS14) related to ‘monoterpenoid biosynthesis’ were down regulated in two cultivars under Pb stress. Terpene synthases (TPSs) play a crucial role during the production of volatile terpenoid. The increased expression of TPS genes can significantly improve the activity of terpene synthases [42]. In this study, the inhibition of *TPS14* (comp_16_179341_c1_seq1) and other related genes in both cultivars probably affected the production of monoterpenoid as well as carotenoid. By contrast, *CYP90B1* (comp_31_170916_c2_seq3), which codes for cytochrome P450 that was involved in brassinosteroid biosynthesis, was up regulated by Pb stress in both cultivars. Consistent with our results, Wang et al. [26] found that the pathways predominantly enriched for the up-regulated transcripts were related to the metabolism of cytochrome P450 in radish responding to Pb.

Previous studies showed that the core terpenoid biosynthetic pathways are heavily regulated by light and multiple other external stimuli (such as sugar and hormone) at gene transcript and posttranslational levels. In addition, the variations in redox state also directly affect the related enzymes [40]. In present study, for the first time, we report that Pb can influence metabolism of terpenoids. This was not surprising, since it was suggested that Pb could cause oxidative damage by inducing the generation of excess of free radicals and reactive oxygen species, and therefore destroy the redox state balance.

In this study, it was interesting to observe that there were profound differences among the DEGs involved in metabolism of terpenoids between the two cultivars after 48 h of Pb treatment. For instance, in AST7001, which can accumulate large amount of Pb, two genes related to ‘limonene and pinene degradation’ were significantly up-regulated. Whereas in Pb tolerant Silverado, *crtB* related to ‘carotenoid biosynthesis’ and *cytokinin dehydrogenase* gene involved in ‘zeatin biosynthesis’ were both significantly up-regulated, and one gene related to ‘biosynthesis of ansamycins’, the only enriched pathway involved in ‘metabolism of polyketides’, was remarkably down regulated (Additional file 6). Polyketides, a subset of the phenylpropanoids, are another large group of secondary metabolites generated by plants. It was reported that phenylpropanoid pathway metabolites of yellow lupine (*Lupinus luteus* L.) roots could promote lead stress tolerance [43]. These differences probably contributed to the variations of Pb accumulation and tolerance between the two cultivars. However, further investigation should be carried out to confirm this hypothesis.

### The expression pattern of genes involved in transport and catabolism

The assembled transcriptome of Pb treatment leaf samples indicated that a total of 21 unigenes related to pathway of ‘transport and catabolism’ were differentially expressed under Pb stress in both cultivars (Additional file 7). Among the unigenes, *HSPA1_8*, *CHMP5* and *CTSB* that were involved in ‘endocytosis’ or ‘lysosome’ were significantly up regulated in both tall fescue cultivars. At the same time, four unigenes related to ‘phagosome’ were only significantly up-regulated in AST7001, including *CALR*, *STX18*, *TUBA* and *TUBB*.

Over the last three decades, there have been evidence indicating that coated pit/coated vesicle-mediated endocytosis resulted in the deposits of heavy metals lanthanum and Pb in maize subsurface cells in the root cap [44]. Recent study demonstrated that rare earth elements can enter the cells via endocytosis in plant cells [45]. Using laser scanning confocal microscope, Wang et al [46] found that cadmium stress dramatically inhibited endocytosis and disrupted the endomembrane organelles, during *Picea wilsonii* pollen germination and tube growth. However, no study has reported on the expression patterns of genes involved in endocytosis under heavy metal stress. In this study, endocytosis related gene *HSPA1_8* (comp_16_151268_c0_seq2) was induced in both cultivars after 48 h of Pb stress, suggesting the common stress response mechanism. On the contrary, *CHMP5* (comp_31_154146_c0_seq6) coding for charged multivesicular body protein 5 was increased in AST7001, but decreased in Silverado, after 48 h of Pb treatment. Previously, CHMP5 was rigorously studied in animal cells, which is essential for late endosome function [47], and plays important roles in programmed cell death in leukemic cells [48]. Although the function of CHMP5 in plant remains unclear, its expression profile probably reflected the different endocytosis response of two tall fescue cultivars with different Pb accumulation ability and tolerance.

Lysosomes are acidic cellular organelles which encapsulate diverse enzymes involved in digestion of extracellular or intracellular macromolecules. It was pointed out that lysosomes related organelles are important mediators in metal homeostasis [49]. In this study, two (*CTSB*, comp_31_178779_c0_seq1; and *AP4S1*, comp_31_148772_c0_seq1) and one unigene (*LGMN,* comp_31_164065_c1_seq1) related to lysosome were up regulated by 48 h of Pb stress in AST7001 and Silverado, respectively, suggesting that tall fescue responded to Pb treatment at transcript level in an attempt control metal homeostasis.

In plant cells, peroxisomes are considered as the major cellular source of various signaling molecules. In addition, the generation of superoxide radicals (O2 ˙-), the presence of catalase and metalloenzyme superoxide dismutase has also been reported in peroxisomes from plant origin [50]. In present study, after Pb treatment, three (*MPV17*, comp_31_169494_c0_seq3; *katE*, comp_31_168938_c1_seq1; and *ACAA1*, comp_16_177871_c0_seq1) and 2 unigenes (*ACAA1*, comp_16_177871_c0_seq1; *katE*, comp_31_167344_c0_seq1) were significantly induced in Silverado after 4 h and 48 h respectively. Simultaneously, in AST7001, two unigenes (*MPV17*, comp_31_151353_c1_seq1; *ACSL*, comp_31_167286_c0_seq2) were up-regulated at 4 h. At 48 h of Pb stress, *PEX16* (comp_31_153781_c0_seq5) and *AGXT* (comp_16_182268_c1_seq1) were up- and down-regulated, respectively (Additional file 6). Therefore, the Pb toxicity may affect the peroxisomal dynamics and destroy the redox homeostasis in tall fescue. The variation of gene transcription probably reflected the different level of oxidative damage and the antioxidant ability between two cultivars [15]. Similarly, cadmium treatment of Arabidopsis could result in time course-dependent changes in peroxisomal dynamics, and affect the ROS metabolism as well as the movement speed of peroxisomes [50]. Arsenic stress can induce the production of peroxule through modulation by peroxin 11a (*PEX11a*) [51]. Our study showed that *PEX16* was also regulated by Pb in AST7001, but its specific role is yet to be discovered.

It was striking that the transcript of four phagosome related unigenes, including *STX18* (comp_31_167390_c1_seq5), *TUBA* (comp_16_173055_c0_seq1), *TUBB* (comp_16_171879_c0_seq3) and *CALR* (comp_31_170482_c0_seq1), was significantly increased under Pb treatment in AST7001 (Additional file 7). However, none of them was up regulated in Silverado.

Syntaxins are a large protein family involved in vesicle sorting, targeting and fusion of the secretory pathway [52]. Several syntaxin homologues had been identified in Arabidopsis, indicating that protein trafficking through the secretory pathway also exists in plant cells [53]. These proteins are considered to take part in vesicle trafficking [54], and each of them might be related to separate steps of the secretory system [55]. For example, *AtPEP12p*, an Arabidopsis syntaxin homologue, was believed to play a role in transport to the vacuole in plants. In AST7001, *STX18* was significantly up regulated under Pb stress. Although its precise function in tall fescue remains unclear, it may participate in vacuolar transport of Pb, since AST7001 owned higher Pb accumulation ability.

Alpha-tubulin proteins are a component of the microtubules, which were considered to be associated with the heavy metal tolerance in insects [56]. Recent report indicated that the transcript level of a-tubulin in heavy metal-tolerant Anopheles mosquitoes was significantly higher under cadmium treatment than normal condition [57]. Although no study has reported on the relationship between tubulin and the heavy metal tolerance in plant, the up regulation of both *TUBA* (coding for tubulin alpha) and *TUBB* (coding for tubulin beta) exclusively in AST7001 is probably associated with the higher Pb accumulation capacity, since AST7001 can uptake and accumulate much more Pb than Silverado [15].

Calreticulin (CRT) is a Ca^2+^-binding protein mainly present in the endoplasmic reticulum of higher plants. It can act as a chaperon or modulator of Ca^2+^ signaling in ER, playing important role in growth and development, as well as in plant responses to diverse stresses. It had been revealed that calreticulin can modulate cold stress through phosphorylation and signaling in rice, on the other hand, over expression of *CRT* increased the rice cold tolerance [58]. Jia et al [59] indicated that under drought treatment, the transcript of wheat *CRT3* was induced in roots, leaves and seedlings. Moreover, over expression of wheat *CRT3* led to the significant improvement of drought tolerance in tobacco. It seemed that the increased expression of *CRT* was positively related to various stress tolerance. In this study, however, calreticulin encoding gene *CALR* showed higher transcript level only in Pb sensitive AST7001 but could accumulate higher concentration of Pb [15]. Therefore, this gene was more likely to be implicated in Pb accumulation than Pb tolerance.

## Conclusions

In summary, this study pioneered in providing the first large-scale transcriptome dataset in tall fescue in response to Pb stress. Totally, 810,146 assembled unique transcripts representing 25,415 unigenes were identified. From which, 3,696 DEGs were found to respond to Pb stress. The expression profiles of selected DEGs were further confirmed by qRT-PCR. In addition, we reported exclusively that the genes involved in the ‘metabolism of terpenoids and polyketides’ and ‘transport and catabolism’ were greatly affected by Pb in tall fescue by GO and pathway enrichment analysis. Furthermore, multiple genes that were implicated in metal homeostasis, antioxidant process, as well as secretory pathway, were found to be remarkably differentially expressed between two tall fescue cultivars. These differences may contribute to the variance in Pb accumulation ability or tolerance between the two cultivars, which provided valuable genomic information and shed new light for further investigation of the molecular mechanism in response to Pb stress in tall fescue or other turf grass species.

## Methods

### Plant materials and growth conditions

During previous studies, we identified two tall fescue cultivars with distinct Pb tolerance, i.e., Pb-tolerant Silverado and Pb-sensitive AST7001. Genotype AST7001 is a turf-type variety with thin leaves and slim stems, developed from turf plot selections in Pennsylvania and Oregon, USA. While Silverado’ (PI 548795) was developed by Pure-Seed Testing, Inc., of Hubbard, USA, and released as an improved turf-type cultivar with a dwarf growth habit. The seeds of both cultivars were obtained from the United States Department of Agriculture, and sown in plastic pots (13 cm diameter, 11 cm deep) filled with sieving sand. The pots were constantly irrigated with water until germination occured. Subsequently, the seedlings were watered daily and fertilized weekly with half strength Hoagland’s solution. The grass height was maintained at 9 cm by clipping. After 50-day of establishment, the grass with uniform growth was transplanted into 300-mL Erlenmeyer flasks filled with half strength Hoagland’s solution [60], after rinsing the roots completely with distilled water. The flask was wrapped with aluminum foil to avoid the light rays. The plants were acclimated for a week in growth chambers (HP300GS-C; Ruihua Instrument, Wuhan, China), with a 14-h photoperiod, photosynthetically active radiation at 300 μmol m^−2^ s^−1^ at the canopy level, a day/night temperature of 24/20 °C, and 75% relative humidity.

### Lead treatment and experimental design

After 1 week of hydroculture, all plants of each cultivar were divided into two groups based on similar transpiration rate following the method described by Hu et al. [61]. One group was transferred to a fresh half strength Hoagland’s solution (CK), while the other was moved into the identical solution but containing 1,000 mg L^−1^ Pb(NO_3_)_2_ (Pb treatment). To avoid possible precipitation reaction caused by Pb, the phosphonium ion strength was reduced to one-tenth of the original recipe. In addition, 0.1 mM of CaO_2_ was supplemented to the solution to provide additional oxygen. All the materials were arranged in a randomized complete block design with multiple independent replicates. According to previous study, the time points of Pb treatment were determined as 4 h and 48 h after treatment. From both the control and Pb treatment groups of each cultivar, leaf samples were collected for Illumina deep sequencing (two biological replicates) and qRT-PCR validation (three biological replicates), respectively. The samples were frozen immediately in liquid nitrogen, and then stored at −80 °C for further analysis.

### Total RNA, mRNA purification, and library preparation

Total RNA was extracted from each sample using Trizol reagent (Invitrogen, Carlsbad, CA) and purified using the RNeasy Plant Mini Kit (Qiagen, Valencia, CA) according to the operation manual. The quantity and quality of total RNA was examined with NanoDrop 8000 spectrophotometer (NanoDrop, Wilmington, DE) and agarose gel electrophoresis. mRNA was then purified from equal amount of total RNA using biotin-Oligo (dT) magnetic beads (Illumina, San Diego, CA), and henceforth cleaved into small fragments using TruSeq RNA Sample Prep Kit, according to the manufacturer’s recommendations. The fragmented mRNA was used to synthesize first-strand cDNA with reverse transcriptase and random primers. Second strand cDNA synthesis was subsequently performed by using DNA Polymerase I and RNase H. The double stranded cDNA was then processed by end reparation, adding a single “A” base to 3’ end, and ligation of the adaptors. After removing the additional free adaptors and mRNA fragments with AMPureXP beads, 200-bp cDNA fragments were enriched by PCR. The enriched cDNA libraries were quantified with Pico green (Quant-iT PicoGreen dsDNA Assay Kit, Invitrogen, P7589) and fluorospectrophotometer (Quantifluor-ST fluorometer, Promega, E6090). Subsequently, their quality was examined with Agilent 2100 Bioanalyzer (Agilent Technologies, Palo Alto, CA). Afterwards, the libraries were sequenced on an Illumina Hiseq 2000 platform in Shanghai Personal Biotechnology Co., Ltd. Analyzer deep sequencing and 6 G data performed for each sample.

### Processing and assembling of Illumina reads

The raw data of RNA-Seq were initially processed by removing the adaptor sequences, reads containing ploy-N or low-quality bases, and reads less than 50 bp. The high quality clean reads were then obtained and used for downstream analysis. By using Trinity software (http://trinityrnaseq.github.io), the clean reads were de novo assembled into contigs and transcripts. The abundance of each transcript was normalized with RPKM [62].

### Unigene functional annotation and classification

Each transcript obtained by Trinity was used to search against the Non Redundant (NR), by NCBI BLAST. Unigenes were obtained based on clustering of tophit result of BLASTX. By searching BLASTx with an E value cutoff of 10^−5^, the unigenes were then assigned to functional categories in KEGG and egg-NOG protein database of monocotyledon Gramineae species including Brachypodium hereafter, rice, maize, wheat and sorghum, In addition, functionally annotated was performed by Blast2GO Gene Ontology functional annotation suit (E-value < 10^−5^) (http://www.blast2go.com/).

### Differential expressed unigenes analysis

The clean read counts aligned to unigene were calculated for each sequenced library. Differential expression analysis of two samples was performed using the DEGseq (http://www-huber.embl.de/users/anders/DESeq). *P*-value < 0.05 and expression changes above 2-fold was set as the threshold to determine the DEGs between different samples.

### GO and KEGG pathway enrichment analysis

The GO terms describe gene function and may occur in different quantities due to changing conditions. It is crucial to find which GO term is significantly overrepresented after stress. On the other hand, KEGG is a database resource for understanding high-level functions and utilities of the biological system, especially large-scale datasets resulting from genome sequencing or other high-throughput experimental methods. In this study, GO and pathway enrichment analysis were employed to identify significantly enriched functional category or metabolic pathways in DEGs. The GO enrichment analysis of the DEGs was carried out by BLAST2GO (http://www.blast2go.com), based on hyper-geometric distribution calculation. At the same time, KOBAS software was used to analyze the statistical enrichment of DEGs in KEGG pathways [63].

### Cluster analysis of DEGs

Hierarchical clustering analysis was performed for differentially expressed unigenes from both tall fescue cultivars. The RPKM counts for each unigene were clustered using the software Cluster 3.0, and JAVA Treeview was applied to view the cluster image. Finally, JAVA Treeview was used to visualize the results [64].

### Validation of RNA-seq data by qRT-PCR

To validate the expression of the target genes, qRT-PCR was carried out using the method described recently by Hu et al. [27]. An *YT-521B* which encodes for an YT521-B-like protein family protein that is ubiquitously expressed in eukaryotic cell was used as a reference gene [65]. Briefly, the first-strand cDNA was synthesized from equal amount of total RNA and used as templates. The 20 μL reaction system was prepared with SYBR Green Real-Time PCR Master Mix (Toyobo, Osaka, Japan). The qPCR was performed on ABI StepOne Plus Real-Time PCR system (Applied Biosystems, Foster City, CA). To ensure product specificity, the melting curve was analyzed for each amplification products at the end of PCR reaction. At the same time, the product size was also examined on agarose gel. The primers used in this study were listed in Table 2. The experiments were repeated twice using three biological replicates. The 2 ^− ΔΔCT^ method was employed to determine the gene changes based on normalization with the reference gene [66], and one-way ANOVA was used to analyze differences between Pb treatments and controls. In addition, to compare the gene expression levels measured by RNA-Seq and qRT-PCR, Pearson correlations were calculated using SPSS 16.0.

## Additional files

Additional file 1: All the unigenes assembled in two tall fescue cultivars. (XLS 38862 kb)
Additional file 2: All the primers used in qRT-PCR. (XLS 19 kb)
Additional file 3: GO enrichment analysis of DEGs for both tall fescue cultivars after treatment with Pb, compared to their controls, respectively. The red thread indicated p-value = 0.05. (TIF 2034 kb)
Additional file 4: KEGG pathway enrichment analysis of DEGs for both tall fescue cultivars after treatment with Pb, compared to their controls, respectively. The red thread indicated p-value = 0.05. (TIF 5381 kb)
Additional file 5: Gene annotation and expression analysis in KEGG pathways of photosynthesis, photosynthesis-antenna proteins, carbon fixation in photosynthetic organisms, methane metabolism and nitrogen metabolism. (XLS 38 kb)
Additional file 6: Gene annotation and expression analysis in metabolism of terpenoids and polyketides. (XLS 25 kb)
Additional file 7: Gene annotation and expression analysis in transport and catabolism metabolism. (XLS 23 kb)

## Abbreviations

BLAST: Basic local alignment search tool
CHMP5: Multivesicular body protein 5
COG: Clusters of Orthologous Groups
CRT: Calreticulin
DEGs: Differentially expressed genes
GO: Gene ontology
KEGG: The Kyotoencyclopedia of genes and genomes database
LHC: Chlorophyll protein complexes
MFE: Minimum free energy
miRNA: MicroRNA
NCBI: National center for biotechnology information
NR: NCBI non-redundant protein database
Nt: Nucleotide sequences database
PERL: Practicalextraction and report language
Pfam: Protein family, Swiss-Prot, a manually annotated and reviewed protein sequence database
qRT-PCR: Quantitative reverse transcription PCR
RNA-Seq: RNA-sequencing
ROS: Reactive oxygen species
RPKM: Reads per kb per million reads
