# Supplementary figures and images for: Transcriptome profilings of two tall fescue (Festuca arundinacea) cultivars in response to lead (Pb) stress
Source: BMC Genomics. 2017 Feb 10;18:145. doi: 10.1186/s12864-016-3479-3 (PMC5301350; doi:10.1186/s12864-016-3479-3)

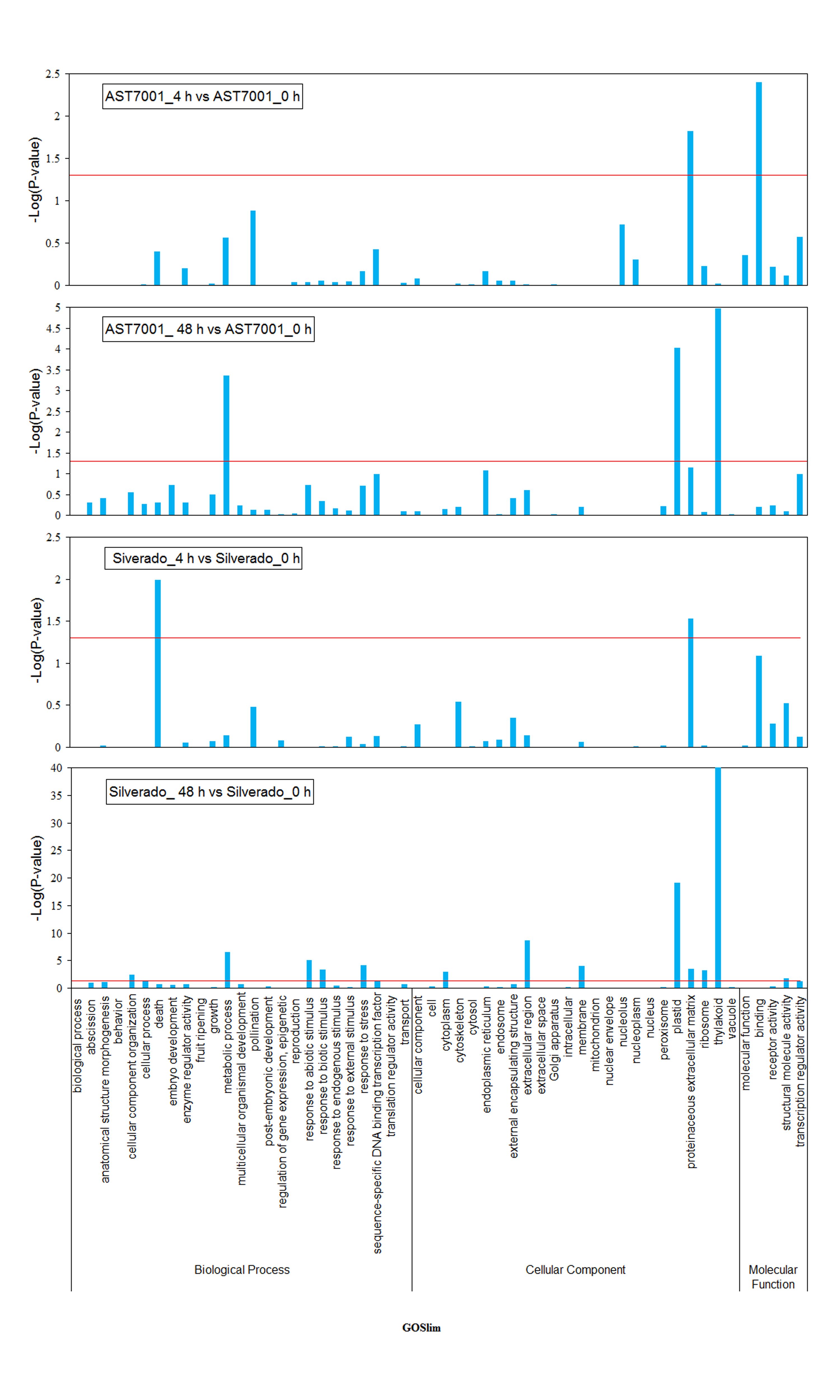

Supplement: Additional file 3: — GO enrichment analysis of DEGs for both tall fescue cultivars after treatment with Pb, compared to their controls, respectively. The red thread indicated p-value = 0.05. (TIF 2034 kb) [file 12864_2016_3479_MOESM3_ESM.tif]

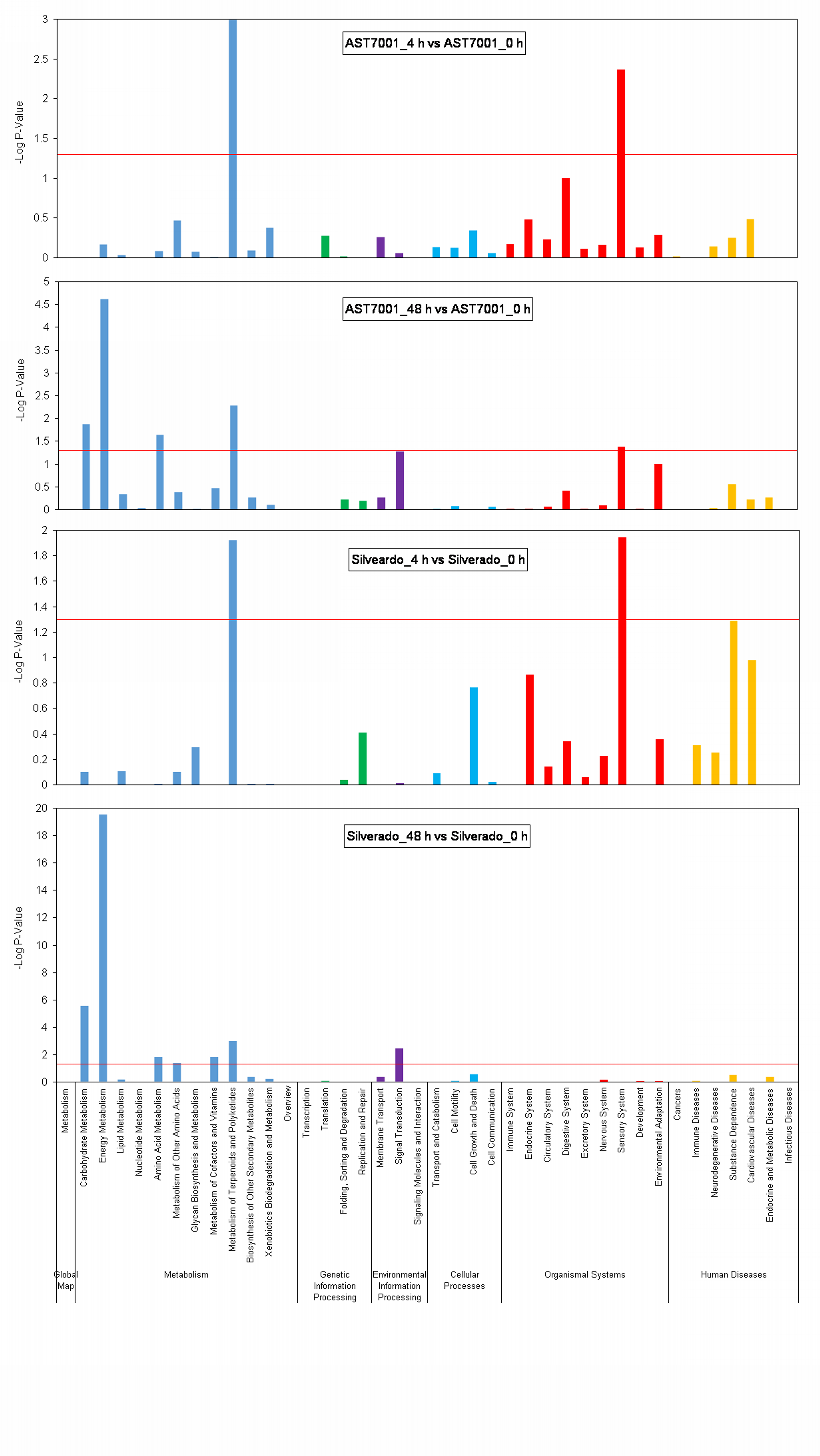

Supplement: Additional file 4: — KEGG pathway enrichment analysis of DEGs for both tall fescue cultivars after treatment with Pb, compared to their controls, respectively. The red thread indicated p-value = 0.05. (TIF 5381 kb) [file 12864_2016_3479_MOESM4_ESM.tif]
